# The risk of osteoporotic fractures after gastrectomy: Findings from the Korean national sample cohort database (2002-2019)

**DOI:** 10.3389/fonc.2022.1014817

**Published:** 2022-11-25

**Authors:** Il Yun, Kyungduk Hurh, Sung Hoon Jeong, Eun-Cheol Park, Sung-In Jang

**Affiliations:** ^1^ Department of Public Health, Graduate School, Yonsei University, Seoul, South Korea; ^2^ Institute of Health Services Research, Yonsei University, Seoul, South Korea; ^3^ Department of Preventive Medicine, Yonsei University College of Medicine, Seoul, South Korea

**Keywords:** gastric cancer, gastrectomy, metabolic bone disease, osteoporotic fracture, cohort study

## Abstract

**Objective:**

This study used a national sample cohort database to investigate the risk of osteoporotic fractures after gastrectomy is performed for gastric cancer.

**Materials and Methods:**

We used data from the Korea National Health Insurance Service-National Sample Cohort between 2002 and 2019. After performing 1:3 propensity score matching, 28,328 individuals were analysed in the final study (7, 082 cases; 21, 246 controls). Gastrectomy data were extracted from the coded claims data, and osteoporotic fractures were defined as the occurrence of fractures in any of the vertebrae, distal radius, humerus, or hip, according to the 10th version of the International Classification of Diseases. A Cox proportional hazards regression model was generated to investigate the association between gastrectomy and risk of osteoporotic fractures.

**Results:**

Patients with gastric cancer who underwent a gastrectomy had a higher risk of osteoporotic fractures as compared to the general population (men, hazard ratio [HR]: 1.13, 95% confidence interval [CI]: 1.00-1.27; women, HR: 1.18, 95% CI: 1.06-1.30). A significantly higher risk of osteoporotic fractures was observed with surgical resection than with endoscopic resection (men, surgical, HR: 1.28, 95% CI: 1.08-1.52, endoscopic, HR: 1.04, 95% CI: 0.90-1.21; women, surgical, HR: 1.34, 95% CI: 1.11-1.62, endoscopic, HR: 1.13, 95% CI: 1.01-1.27). In men, the risk of hip fracture was the highest among the four fracture sites (HR: 1.18, 95% CI:0.89-1.56), while in women, the risk of vertebral fracture after gastrectomy was the highest (HR: 1.16, 95% CI: 0.99-1.35).

**Conclusion:**

Patients with gastric cancer who underwent gastrectomy had a higher risk of osteoporotic fractures as compared to the general population. This suggests the need for bone metabolism management in patients with gastric cancer to prevent post-gastrectomy complications.

## Introduction

Gastric cancer is the fourth most common cancer worldwide (1) and has been the most common cancer in South Korea since 1999, when the Korea Central Cancer Registry first reported nationwide cancer incidence data (2). Fortunately, through early detection and curative treatment such as gastrectomy, the long-term survival rates increase considerably (3). In South Korea, early diagnosis of gastric cancer leads to a survival rate of over 90% (4). However, the complications associated with gastrectomy cannot be overlooked. Osteoporosis and fractures commonly occur after gastrectomy is performed for gastric cancer (5, 6). Previous studies have reported an incidence of 32-42% for osteoporosis after gastrectomy, and approximately 40% for fractures (7–9). In addition, osteoporotic fractures have been found to increase the socioeconomic burden on individuals and society (10) as well as impair the quality of life (11).

Although several studies have reported a high prevalence of metabolic bone diseases such as osteomalacia and osteoporosis after gastrectomy was performed for gastric cancer (7, 12) a systematic post-surgery management program has not yet been established. While the incidence of osteoporotic fractures is expected to increase due to gastrectomy, the exact incidence has not been confirmed because the hospitals wherein the gastrectomies are performed are usually different from those that diagnose osteoporotic fractures (7). Therefore, longitudinal observational studies on the risk of new-onset osteoporotic fractures have rarely been conducted in South Korea.

The current study aimed to identify the risk of osteoporotic fractures after gastrectomy for gastric cancer using a national sample cohort database. Furthermore, based on the results of this study, we aimed to provide evidence of the need for systematic management to prevent complications after gastrectomy.

## Materials and methods

### Data and study population

This population-based cohort study analysed data from the Korea National Health Insurance Service-National Sample Cohort (NHIS-NSC) database between 2002 and 2019. Since the introduction of universal health coverage in 1989, all South Koreans have been obliged to subscribe to the NHIS; thus, approximately 98% of the total population has been enrolled. The NHIS-NSC database contains data on all cases of Korean healthcare utilisation and information on the sociodemographic characteristics and diagnosis codes as per the International Classification of Diseases, 10^th^ revision (ICD-10). The study protocol was approved by the Institutional Review Board of Yonsei University Health System (IRB Number: Y-2020-0031). The requirement for informed consent was waived owing to the retrospective nature of the study and the lack of any identifiable information in the data.

Of the 47,851,928 individuals in the NHIS-NSC database as of 2002, 46,605,433 individuals were included in the sample cohort after excluding non-citizens. After random sampling, the database included 1,025,340 individuals, accounting for 2.2% of the total population of South Korea (13). The years 2002 and 2003 were designated as washout periods since the first 2-year might be the consequence of pre-existing diseases and the outcome of our study was the newly onset osteoporotic fractures. Moreover, to avoid reverse causality, we excluded those individuals who were found to have osteoporotic fractures within one year after gastrectomy. We then performed 1:3 propensity score matching (matching variables: sex, age, and medical insurance) to include the normal general population who did not undergo gastrectomy as the control group. Consequently, 28,328 individuals in total were analysed in the final study, with 7,082 in the case group and 21, 246 in the control study.

### Variables

The dependent variable was the risk of osteoporotic fractures, which was defined as the occurrence of fractures in any of the vertebrae (ICD-10 codes S220, S221, S320, M484, and M485), distal radius (ICD-10 codes S525 and S526), humerus (ICD-10 codes S422 and S423), or hip (ICD-10 codes S720 and S721) (14, 15). The main variable of interest was gastrectomy, which included both endoscopic and surgical resections. The date of diagnosis of any of the four types of osteoporotic fractures or the last date of the study period (30 December 2019) was set as the final follow-up date (16).

As covariates, we included sociodemographic variables such as age, medical insurance, income, region, and health status-related variables such as the Charlson Comorbidity Index (CCI), hypertension, and diabetes mellitus. The Korean National Health Insurance System covers the entire population residing within the territory of the Republic of Korea, except for beneficiaries of medical aid. Additionally, the Korean government offers a medical aid program to those who cannot afford to pay for healthcare coverage (17). Taking this into account, medical insurance was classified into three categories i.e., regionally-insured, workplace-insured, and medical aid. Regions were defined using the position value for the relative comparison (PARC) index, which has been widely used to diagnose the level of medical care by region in Korea (18, 19). PARC is an objective indicator used to determine the relative position of medical care in a region in terms of demand, supply, access, use, and quality compared to other regions (18). The PARC value ranges from -1 to 1, the best being 1, the average indicated by 0, and the worst being -1 (19). A PARC value less than -0.33 was classified as a medically vulnerable region. The CCI score is an index for evaluating patient comorbidities, which can be calculated by assigning weights of 1-6 points for 19 comorbidities. Categories of comorbid diseases included in the CCI score consisted of myocardial, vascular, pulmonary, endocrine kidney, gastrointestinal, cancer, immune, and neurologic comorbidities (20). ICD-10 diagnostic codes were used to calculate the CCI scores of the study population for each comorbid disease (21), and CCI scores were classified into two categories i.e., approximately 0-2, and ≥ 3.

### Statistical analysis

Chi-square tests were performed to investigate the general characteristics of the study population. A Cox proportional hazards model was generated to examine the association between gastrectomy and the risk of osteoporotic fractures. Kaplan–Meier survival curves were used to compare survival probabilities between the groups, and a stratified log-rank test was used to compare the Kaplan–Meier curves of the matched cohort (22, 23). The adjusted hazard ratios (HRs) with 95% confidence intervals (CIs) were presented as the key results. For all analyses, we used SAS software (version 9.4; SAS Institute Inc., Cary, NC, USA). Statistical significance was set at *p* <.05.

## Results

**Table 1** presents the general characteristics of the study population after propensity score matching in a 1:3 ratio. Among the 28,328 individuals eligible for analysis, 7,082 were part of the case group of gastric cancer patients who underwent gastrectomy, and the remaining 21,246 were part of the control group recruited from the general population. Osteoporotic fractures were found in 1,415 (8.4%) of the 16,904 men and 1,909 (16.7%) of the 11,424 women.

**Table 1:** General characteristics of the study population after 1:3 propensity score matching.

Variables	Total	Men (n = 16,904)				*p*-value	Women (n = 11,424)				*p*-value
		Osteoporotic fractures					Osteoporotic fractures				
		Yes (n = 1,415)		No (n = 15,489)			Yes (n = 1,909)		No (n = 9,515)		
	n	n	%	n	%		n	%	n	%	
**Study Population**						0.077					0.066
Case	7,082	382	9.04	3,844	90.96		509	17.82	2,347	82.18	
Control	21,246	1,033	8.15	11,645	91.85		1,400	16.34	7,168	83.66	
**Age**						<.0001					<.0001
20-29	232	3	3.95	73	96.05		4	2.56	152	97.44	
30-39	1,208	12	2.29	512	97.71		5	0.73	679	99.27	
40-49	3,052	49	3.17	1,495	96.83		56	3.71	1,452	96.29	
50-59	6,096	179	4.92	3,461	95.08		239	9.73	2,217	90.27	
60-69	8,616	418	7.61	5,078	92.39		550	17.63	2,570	82.37	
≥ 70	9,124	754	13.41	4,870	86.59		1,055	30.14	2,445	69.86	
**Medical Insurance**						0.001					0.001
Regionally-Insured	9,036	446	8.16	5,022	91.84		567	15.89	3,001	84.11	
Workplace-insured	18,888	938	8.35	10,302	91.65		1,289	16.85	6,359	83.15	
Medical Aid	404	31	15.82	165	84.18		53	25.48	155	74.52	
**Income**						0.147					0.002
Low	6,671	346	9.14	3,438	90.86		479	16.59	2,408	83.41	
Medium	9,591	472	8.10	5,356	91.90		571	15.17	3,192	84.83	
High	12,066	597	8.19	6,695	91.81		859	17.99	3,915	82.01	
**Region**						<.0001					<.0001
Vulnerable area	3,792	274	11.68	2,072	88.32		324	22.41	1,122	77.59	
Non-vulnerable area	24,536	1,141	7.84	13,417	92.16		1,585	15.88	8,393	84.12	
**Charlson Comorbidity Index**						<.0001					<.0001
0 ~ 2	7,857	246	5.11	4,572	94.89		320	10.53	2,719	89.47	
≥ 3	20,471	1,169	9.67	10,917	90.33		1,589	18.95	6,796	81.05	
**Hypertension**						0.069					<.0001
Yes	2,327	136	9.67	1,270	90.33		239	25.95	682	74.05	
No	26,001	1,279	8.25	14,219	91.75		1,670	15.90	8,833	84.10	
**Diabetes mellitus**						0.900					<.0001
Yes	982	57	8.52	612	91.48		92	29.39	221	70.61	
No	27,346	1,358	8.36	14,877	91.64		1,817	16.35	9,294	83.65	

**Table 2** shows the results of a Cox proportional hazards regression analysis of the association between gastrectomy and the risk of osteoporotic fractures after controlling for all covariates. Patients with gastric cancer who underwent gastrectomy in the case group had a higher risk of osteoporotic fractures than the general population in the control group (men, HR: 1.13, 95% CI:1.00-1.27; women, HR: 1.18, 95% CI: 1.06-1.30). Among covariates, medical insurance (women, regionally insured, HR: 1.19, 95% CI: 1.08-1.32), income (men, low, HR: 1.19, 95% CI: 1.04-1.36), region (men, vulnerable area, HR: 1.24, 95% CI: 1.08-1.41), and CCI score (men, ≥3, HR: 1.33, 95% CI: 1.15-1.54; women, ≥3, HR: 1.16, 95% CI: 1.03-1.32) were found to be statistically significant factors influencing the risk of osteoporotic fractures.

**Table 2:** Results of Cox proportional hazard regression analysis on the association between gastrectomy and risk of osteoporotic fractures.

Variables	Men				Women			
	Risk of osteoporotic fractures				Risk of osteoporotic fractures			
	Adjusted HR	95% CI			Adjusted HR	95% CI		
**Study Population**								
Case	1.13	(1.00	-	1.27)	1.18	(1.06	-	1.30)
Control	1.00				1.00			
**Age**								
20-29	1.00				1.00			
30-39	0.55	(0.16	-	1.97)	0.26	(0.07	-	0.98)
40-49	0.70	(0.22	-	2.26)	1.25	(0.45	-	3.45)
50-59	0.91	(0.29	-	2.86)	2.76	(1.03	-	7.42)
60-69	1.15	(0.37	-	3.60)	4.41	(1.65	-	11.82)
≥ 70	1.48	(0.47	-	4.61)	5.59	(2.09	-	14.96)
**Medical Insurance**								
Regionally-Insured	1.09	(0.97	-	1.22)	1.19	(1.08	-	1.32)
Workplace-insured	1.00				1.00			
Medical Aid	1.18	(0.81	-	1.71)	1.01	(0.75	-	1.34)
**Income**								
Low	1.19	(1.04	-	1.36)	1.04	(0.92	-	1.17)
Medium	1.08	(0.95	-	1.22)	1.01	(0.90	-	1.12)
High	1.00				1.00			
**Region**								
Vulnerable area	1.24	(1.08	-	1.41)	1.06	(0.94	-	1.20)
Non-vulnerable area	1.00				1.00			
**Charlson Comorbidity Index**								
0 ~ 2	1.00				1.00			
≥ 3	1.33	(1.15	-	1.54)	1.16	(1.03	-	1.32)
**Hypertension**								
Yes	0.91	(0.76	-	1.09)	0.95	(0.82	-	1.09)
No	1.00				1.00			
**Diabetes mellitus**								
Yes	0.89	(0.68	-	1.16)	1.22	(0.99	-	1.51)
No	1.00				1.00			

**Figure 1:**
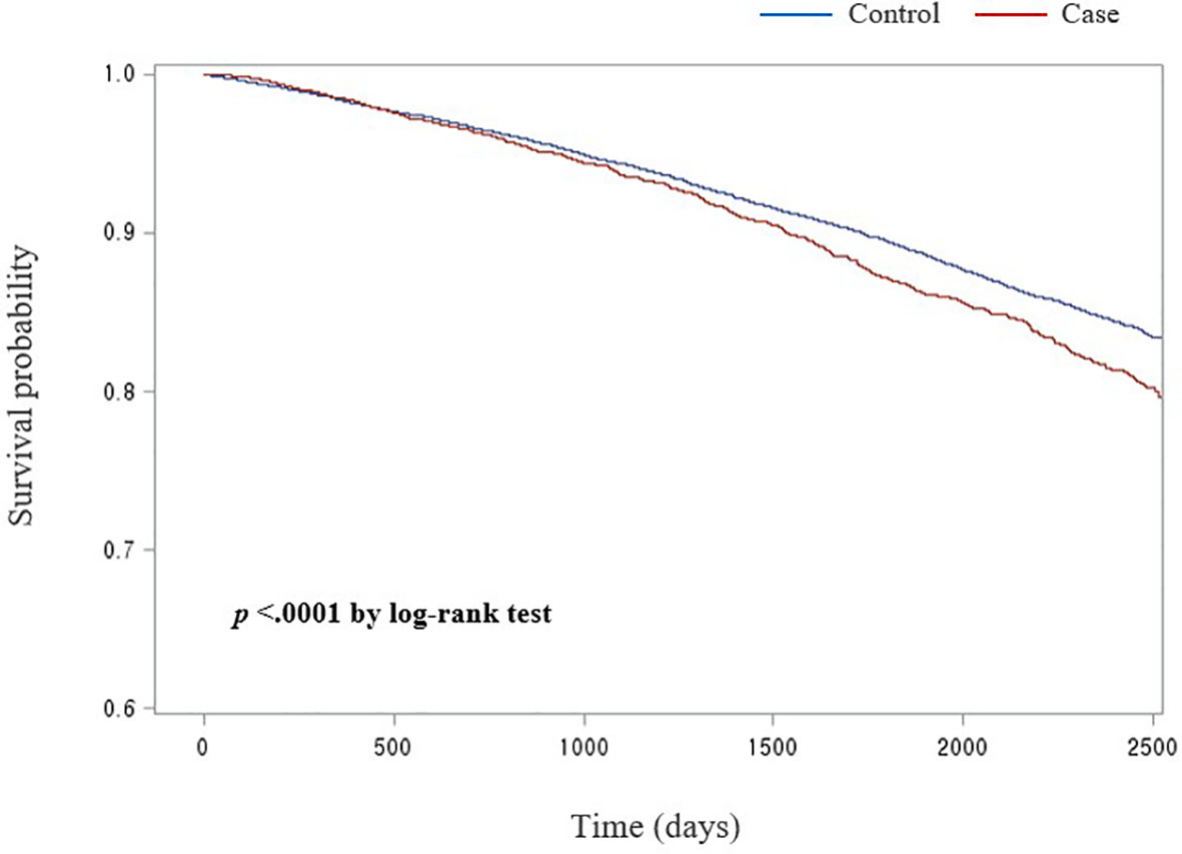
Kaplan-Meier curve for compare survival probabilities between case and control group.

**Figure 1** presents the Kaplan–Meier survival curves. There were difference in the survival probabilities between patients with osteoporotic fractures after gastrectomy and the control groups during the entire follow-up period (*p* <.0001 for log-rank test).

**Figure 2:**
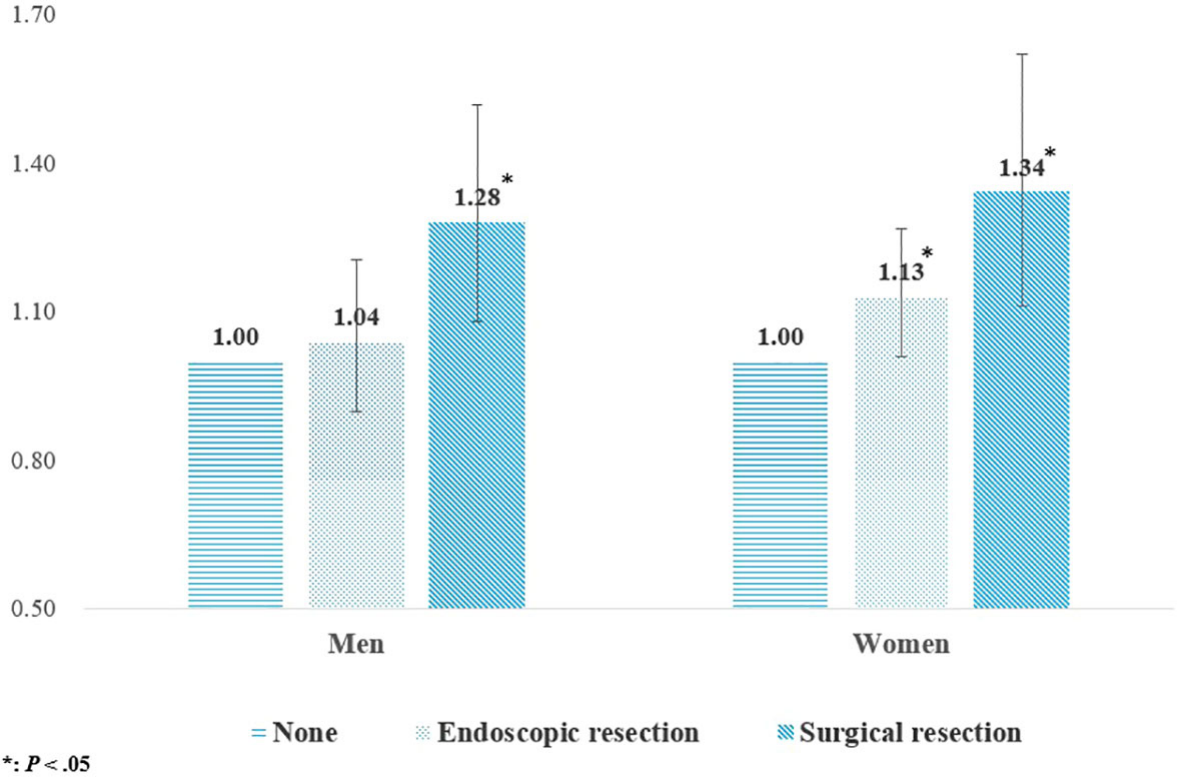
Results of subgroup analysis stratified by gastrectomy method.

Since gastrectomy, which was our main variable of interest, included both endoscopic and surgical resections, we performed a subgroup analysis with stratification by the method of gastrectomy. The results for the same are depicted in **Figure 2**. As compared to the men in the control group, the risk of osteoporotic fractures in men was 1.04 times higher in those who underwent endoscopic resections (95% CI: 0.90-1.21), and 1.28 times higher in those who underwent surgical resection (95% CI: 1.08-1.52). Similarly, compared to women in the control group, the women who underwent endoscopic resection had a 1.13 times higher risk of osteoporotic fractures (95% CI: 1.01-1.27), and those who underwent surgical resection had a 1.34 times higher risk (95% CI: 1.11-1.62).

Subgroup analysis was performed according to the four osteoporotic fractures sites, and the results are shown in **Table 3**. In male patients with gastric cancer who underwent gastrectomy, the risk of hip fracture was the highest among the four fracture sites (HR:1.18, 95% CI: 0.89-1.56). In contrast, in women, the risk of vertebral fracture after gastrectomy was the highest (HR: 1.16, 95% CI: 0.99-1.35).

**Table 3:** Results of subgroup analysis stratified by osteoporotic fractures site.

Men	Risk of osteoporotic fractures																
	None	Vertebral fracture				Distal radius fracture				Humeral fracture				Hip fracture			
	AHR	AHR	95% CI			AHR	95% CI			AHR	95% CI			AHR	95% CI		
**Study Population**																	
Case		1.01	(0.85	-	1.20)	1.06	(0.81	-	1.38)	0.76	(0.43	-	1.35)	1.18	(0.89	-	1.56)
Control	1.00																
**Women**	**Risk of osteoporotic fractures**																
	**None**	**Vertebral fracture**				**Distal radius fracture**				**Humeral fracture**				**Hip fracture**			
	**AHR**	**AHR**	**95% CI**			**AHR**	**95% CI**			**AHR**	**95% CI**			**AHR**	**95% CI**		
**Study Population**																	
Case		1.16	(0.99	-	1.35)	0.84	(0.71	-	1.01)	0.92	(0.62	-	1.38)	1.13	(0.79	-	1.60)
Control	1.00																

## Discussion

It is well established that metabolic bone diseases can occur as late complications in patients with gastric cancer who undergo gastrectomy (24, 25). Previous studies have reported a decrease in the intestinal absorption of calcium and vitamin D as a cause of bone disorders such as osteoporosis and osteomalacia after gastrectomy (12, 26). In addition, it has also been demonstrated that decreased bone mass after gastrectomy increases the cumulative incidence of fractures (6, 27). In south Korea, an increase in the long-term survival rate after gastrectomy in patients with gastric cancer has led to an anticipated rise in metabolic bone diseases and fractures related to gastrectomy (28); however, only a few studies have explored this yet. Thus, this retrospective observational study attempted to investigate the risk of osteoporotic fractures among possible bone diseases after gastrectomy, using a national health insurance claims data.

The present study had three key findings. First, we found that patients with gastric cancer who underwent gastrectomy were at a higher risk of new-onset osteoporotic fractures than the general population. This is consistent with the results of a study that used the nationwide claims data and showed that osteoporosis and osteoporotic fracture incidences were high in patients within a relatively short period of time after gastrectomy in gastric cancer patients who underwent gastrectomy for 3 years (7). Therefore, our findings suggest that the systematic management of metabolic bone diseases and fractures is necessary immediately after gastrectomy. Second, a significantly higher risk of osteoporotic fracture was observed for surgical resection, as compared with endoscopic resection. This finding was similar to that of a previous meta-analysis, which confirmed that compared to surgical resection, endoscopic resection was associated with similar long-term outcomes and considerable advantages in terms of operation time, hospital stay, costs, and complications (29). While endoscopic resections can be performed on patients with relatively early stage gastric cancer, development in medical technology for wide-scale applications will be required to restore function and reduce complications after resection. Third, we found that men had the highest risk of hip fractures and women the highest risk of vertebral fractures among the four fracture sites. Since several studies have demonstrated that hip and vertebral fractures are associated with increased mortality risk (30, 31), bone metabolism management is vital in patients who undergo gastrectomy.

This study had several limitations. First, because we used retrospective cohort data, we could not adjust for any potential confounding factors that could affect the incidence of osteoporotic fractures, such as nutrients, diet, and exercise. Second, diagnostic inaccuracies in the health insurance claims database may limit the accuracy of the diagnostic information (32). To correct for this, we included both primary and secondary diagnostic codes in the analysis (16). In addition, since we only used ICD-10 coded claims data, we could not control for the severity of osteoporotic fractures. Despite these limitations, our study had certain strengths because we used the most up-to-date national sample cohort database. Due to the representativeness of the data, our results can be generalised to the entire population of South Korea as well as to other countries with similar demographic characteristics. Unlike several previous studies focusing mainly on the association between gastrectomy and osteoporosis occurrence (33, 34), there was a clear difference in that we also considered the risk of osteoporotic fractures and resection methods, and preformed 1:3 propensity score matching. Furthermore, the follow-up period of our data was 18 years, making it possible to infer long-term associations.

In conclusion, our study found a significant relationship between gastrectomy and the risk of osteoporotic fractures using data from the South Korean National Sample Cohort database. Patients with gastric cancer who underwent gastrectomy had a higher risk of osteoporotic fractures than the general population, and this tendency was particularly observed when they underwent surgical resection rather than endoscopic resection. These findings highlight the need to systematically manage bone metabolism immediately after gastrectomy in patients with gastric cancer.

## Data availability statement

Publicly available datasets were analyzed in this study. This data can be found here: https://nhiss.nhis.or.kr/bd/ab/bdaba000eng.do.

## Ethics statement

The studies involving human participants were reviewed and approved by the Institutional Review Board of Yonsei University Health System (IRB Number: Y-2020-0031). Written informed consent for participation was not required for this study in accordance with the national legislation and the institutional requirements.

## Author contributions

IY made a substantial contribution to the concept or design of the work. IY and KH contributed to the acquisition, analysis, or interpretation of data. SH, E-CP, and S-IJ drafted the article or revised it critically for important intellectual content. All authors contributed to the article and approved the submitted version.

## Funding

This research was supported by a grant of the Korea Health Technology R&D Project through the Korea Health Industry Development Institute (KHIDI), funded by the Ministry of Health & Welfare, Republic of Korea (grant number: HI20C1130).

## Acknowledgments

We would like to thank the colleagues from the Department of Public Health, Graduate School of Yonsei University provided advice for this manuscript.

## Conflict of interest

The authors declare that the research was conducted in the absence of any commercial or financial relationships that could be construed as a potential conflict of interest.

## Publisher’s note

All claims expressed in this article are solely those of the authors and do not necessarily represent those of their affiliated organizations, or those of the publisher, the editors and the reviewers. Any product that may be evaluated in this article, or claim that may be made by its manufacturer, is not guaranteed or endorsed by the publisher.
